# Replacing Manual Operation with Bio-Automation II: Construction of a Biological Digestion Gene Circuit to Eliminate the Interference of Food Matrices in the Rapid Detection of Heavy Metals

**DOI:** 10.3390/foods14213798

**Published:** 2025-11-06

**Authors:** Shiqi Xia, Shijing Chen, Hongfei Su, Liangshu Hu, Xiaozhe Qi, Mingzhang Guo

**Affiliations:** 1 Key Laboratory of Digital-Intelligence and Dynamic Perception for Food Quality of China Light Industry, Beijing Technology and Business University, Beijing 100048, China; xiashiqi@st.btbu.edu.cn (S.X.); chenshijing@st.btbu.edu.cn (S.C.); suhongfei@st.btbu.edu.cn (H.S.); liangshu_hu@163.com (L.H.); 2 Standards and Quality Center of National Food and Strategic Reserves Administration (NAFRA), Beijing 100834, China

**Keywords:** whole-cell biosensor, food matrix, bio-digestion, Hg^2+^

## Abstract

Food matrices such as phytic acid, starch, and proteins can chelate heavy metals, acting as stabilizers that significantly hinder accurately detecting heavy metal contamination. This study proposes a biological digestion strategy to overcome such interference. The gene sequences for phytase (*appA*) from *Escherichia coli (E. coli)*, α-amylase (*amyA*) from *Escherichia coli (E. coli)*, and protease (*AO090120000474*) from *Aspergillus oryzae* were identified via bioinformatics screening. Whole-cell biosensors were then developed to simultaneously detect mercury ions (Hg^2+^) and digest phytate, starch, and proteins. In the presence of 100 μM Hg^2+^, biosensor responses improved by 1.43-, 1.38-, and 1.11-fold, respectively. A “heavy metal pollutant bio-digestion pathway” was constructed by integrating genes for synthesizing phytic acid, starch, and protein with those for Hg^2+^ detection. In the presence of 100 μM Hg^2+^, the detection effect was improved by 1.36-fold. The detection limit of the BαAP whole-cell biosensor was 0.082 μM, while the limit of quantitation was 0.272 μM. The study effectively addresses the limitations of biosensor performance in real sample detection.

## 1. Introduction

Mercury is considered by World Health Organization (WHO) as one of the top ten chemicals of major public health concern [1]. Exposure to mercury can cause serious health effects, including toxicity to the nervous, digestive, and immune systems, as well as the lungs, kidneys, skin, and eyes [2]. It is particularly hazardous to fetal and early childhood development. Mercury pollution occurs in various forms, including elemental mercury, methylmercury, and both soluble and insoluble inorganic mercury species [3,4,5]. Human exposure primarily occurs through dietary intake of methylmercury-contaminated foods such as fish, shellfish, and rice [6,7]. Among subsistence fishing populations, it is estimated that between 1.5 and 17 out of every 1000 children exhibit cognitive impairment due to methylmercury exposure [8]. Given the persistence of mercury pollution, developing sensitive and practical mercury detection technologies is essential for prevention and control.

Currently, the gold standard for mercury detection relies on precise instrumentation-based methods, such as atomic absorption spectrometry and inductively coupled plasma mass spectrometry [9,10]. Although accurate, these methods require expensive equipment and skilled personnel, making them unsuitable for on-site detection. Recently, biosensors based on aptamers, antibodies, enzymes, and whole cells have emerged as promising tools for rapid, on-site mercury detection [11,12,13], offering improved sensitivity and convenience. Despite these advances, two major bottlenecks limit the practical application of these mercury biosensors in food sample analysis. First, most biosensors can detect Hg^2+^, while quantifying methylmercury or total mercury content is of greater relevance for food safety. Second, current biosensors can detect only free mercury pollutants. Yet, mercury pollutants in food matrices are often complex or encapsulated by macromolecular components such as protein, starch, and phytic acid. Among these, proteins exhibit the strongest chelating ability for mercury pollutants, with some even encapsulating mercury pollutants within their molecular structures [14], potentially resulting in false-negative readings. A conventional solution to these challenges involves the digestion of food matrices and the oxidation of methylmercury into Hg^2+^ through strong acid or microwave digestion [15]. However, these manual pretreatment processes are hazardous, require specialized equipment, and are time-consuming—factors incompatible with principles of rapid, on-site food safety testing, and cannot meet the needs of actual heavy metal pollution supervision.

**Figure 1:**
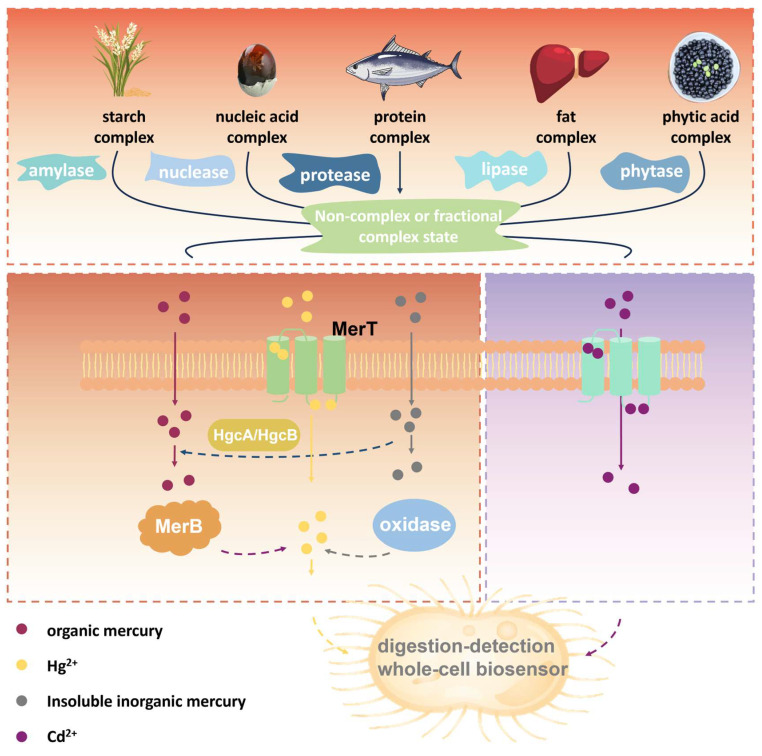
Schematic diagram of biological digestion technology.

In previous research, we proposed a biological digestion strategy (Figure 1), wherein high-efficiency biological enzymes or other biological elements replace physical or chemical digestion processes. These elements are integrated into whole-cell biosensors as gene circuits, thereby reducing the need for manual sample preparation and instrument-dependent procedures [16,17,18]. Compared with strong acid or microwave digestion, biological digestion technology is safer, more environmentally friendly, and better suited for on-site detection. Furthermore, when incorporated into whole-cell biosensors, the biological digestion gene circuits will automatically replicate and express elements via microbial proliferation, without increasing the complexity or cost of biosensor production. Previously, we successfully constructed a biological digestion gene circuit using the methylmercury lyase gene (*merB*), which enabled the conversion of methylmercury into Hg^2+^ and addressed the first bottleneck problem in mercury pollutant biosensing detection [16].

Continuing this work, the present study addresses the second bottleneck by developing gene circuits that enable whole-cell biosensors to enzymatically digest food matrices and release mercury pollutants from macromolecular complexes into a detectable free state. The study begins by evaluating the influence of specific food matrix components on detecting the whole-cell biosensor. Following identification, relevant digestive enzymes are selected, and their genes are retrieved using the KEGG database. Whole-cell biosensors were constructed and tested for relative fluorescence intensity under various environmental conditions. Finally, a tandem gene circuit integrating both digestion and detection pathways was built to mitigate matrix interference and enhance the practical application of mercury biosensors in food samples.

## 2. Materials and Methods

### 2.1. Chemicals and Bacteria

Methylmercury (CH_3_Hg, 98%) and mercury dichloride (HgCl_2_, 99.5%) were purchased from Xiya Reagent (Linyi, China). *E. coli* DH5α (laboratory preservation) was used as the chassis cells for all plasmids constructed in this study. The *E. coli* strain containing the ebMerR-RFP sensor was also derived from laboratory development and preservation. Codon-optimized gene sequences for *amyA, appA,* and *AO090120000474* were synthesized by Ruibio Biotech (Beijing, China), which also performed oligonucleotide synthesis and plasmid sequencing. Cells were cultured in Luria–Bertani (LB) broth containing 5 g/L NaCl, 5 g/L yeast extract, and 10 g/L tryptone. Solid plates were prepared with the same composition supplemented with 1.5% (*w*/*v*) agar. The antibiotics kanamycin (Kan) was added to a final concentration of 50 μg/mL for plasmid selection.

### 2.2. Extraction of Matrix Ingredients from Different Foods

Extraction of fish proteins: Four grams of fish were homogenized with 2 mL of 1.0 mol/L hydrochloric acid, followed by adding 54 mL of distilled water. The pH of the resulting solution was adjusted to 2.39 and shaken for 1.5 h. The mixture was centrifuged at 4500× *g* rpm for 15 min, and the supernatant was collected and centrifuged again under the same conditions. The final supernatant was used as a protein extract.

Extraction of rice starch: Rice flour was passed through a 100-mesh sieve. A 0.5% SDS solution was added at a liquid-to-solid ratio of 6:1, and the mixture was soaked for 2 h. The sample was centrifuged at 4500× *g* rpm for 15 min, and the supernatant was discarded. 50 mL of distilled water was added, and the process was repeated three times. The resulting precipitate was dried at 45 °C for 12 h to obtain the starch extract.

Extraction of black bean phytic acid: Black beans were washed, dried, ground, and sieved through a 0.425 mm mesh. Two grams of the black bean sample were mixed with 32 mL of 10% sodium sulfate-hydrochloric acid solution (pH = 2). The mixture was shaken at 60 °C for 1.5 h, centrifuged at 10,000× *g* rpm for 10 min. The supernatant was collected as the phytic acid extract.

### 2.3. Bioinformatics Analysis of Microbial Matrix Lyase Genes

Gene sequences for microbial amylase, protease, and phytase were obtained from the Kyoto Encyclopedia of Genes and Genomes (KEGG) database. Specifically, *appA* phytase from *E. coli*, *amyA* α-amylase from *E. coli* MG1655, and *AO090120000474* protease from *Aspergillus oryzae* RIB40 were selected for analysis (see Supplementary Table S1).

### 2.4. Construction of a Matrix Digestion Whole-Cell Biosensor

The plasmid backbone was modified with the ebMerR-RFP regulatory module (laboratory stock), which was used for biosensor construction. The amylase, phytase, and protease genes were individually inserted into the plasmid backbone at the BglII and HindIII endonuclease restriction sites to generate single-function matrix digestion whole-cell biosensors (see Supplementary Figures S1–S3). The constructed plasmid was transformed into *E. coli* DH5α, cultured in Luria–Bertani (LB) broth, and stored in 50% glycerol at −80 °C until use.

### 2.5. Construction of a Heavy Metal Pollutant Bio-Digestion Pathway

The plasmid backbone was based on the previously constructed WCB-*amyA* plasmid. The *appA* gene, equipped with a constitutive promoter and terminator, was introduced into the plasmid backbone using the restriction enzyme XbaI. Subsequently, the *AO090120000474* gene, with a constitutive promoter and terminator, was inserted downstream of *appA* using the endonuclease HindIII to create a bio-digestion pathway targeting heavy metal contaminants (see Supplementary Figure S4). The constructed plasmid was transformed into *E. coli* DH5α, cultured in Luria–Bertani (LB) broth, and stored in 50% glycerol at −80 °C until use.

### 2.6. Mercury and Methylmercury Detection Procedures

The synthesized biosensors were activated by overnight incubation in LB broth supplemented with kanamycin. After activation, the overnight culture was diluted to 1% (*v*/*v*) and incubated at 37 °C with shaking at 220 rpm until the optical density at 600 nm (OD_600_) reached approximately 0.6. For detection, 5 mL of activated culture was mixed with 5 mL of fresh LB broth (containing kanamycin), 100 μL of heavy metal solution, and 200 μL of matrix extract in a 50 mL flask. The mixture was incubated at 37 °C and 220 rpm. After incubation, 200 μL of culture was selected to measure OD_600_. Then, 2 mL of culture was centrifuged at 10,000× *g*, the supernatant was discarded, and the precipitate was suspended in 200 μL of 0.9% saline. The entire volume was transferred to a 96-well black microtiter plate for fluorescence measurement. The fluorescence intensity of the solution was measured using a full-length spectral scanner at an excitation wavelength of 587 nm and an emission wavelength of 610 nm (corresponding to the excitation and emission maxima of red fluorescent protein. All experiments were conducted in triplicate. The relative fluorescence intensity was defined as the ratio of fluorescence to OD_600_.

### 2.7. Impact of Environmental Factors on Whole-Cell Biosensors

To assess the influence of external environmental conditions on whole-cell biosensor performance, a 100 μM Hg^2+^ was added to the bacterial cultures with an OD_600_ of 0.6. The mixtures were incubated at 37 °C and 220 rpm for 15, 30, 45, 60, 75, and 90 min, respectively. In addition, to evaluate pH effects, the culture medium was adjusted to pH values of 5.5, 6.5, 7.5, and 8.5, followed by incubation at 37 °C and 220 rpm for 45 min. Temperature effects were investigated by incubating cultures at 20 °C, 25 °C, 30 °C, 37 °C, and 40 °C for 45 min. Following treatment, OD_600_ and fluorescence measurements of the samples were performed using a fluorescent microwell reader.

### 2.8. Biosensor-Based Analysis of Mercury and Methylmercury Chelation State in Fish, Rice, and Black Beans

The samples used in this study included *Larimichthys crocea* meat, rice, and black beans. Fish were cut into small pieces, while rice and black beans were ground into powder and passed through a 20-mesh sieve. Mercury ions and methylmercury were added to the respective samples at varying concentrations, homogenized thoroughly, and incubated for 5 h to facilitate chelation. The resulting samples were then analyzed for mercury ion and methylmercury content using the whole-cell biosensor.

### 2.9. Statistics Analysis

All experiments were conducted in triplicate, and results were reported as the arithmetic mean ± standard deviation (SD).

## 3. Results and Discussion

### 3.1. The Effect of Food Matrix on the Accuracy of Whole-Cell Biosensor for Mercury Detection

**Figure 2:**
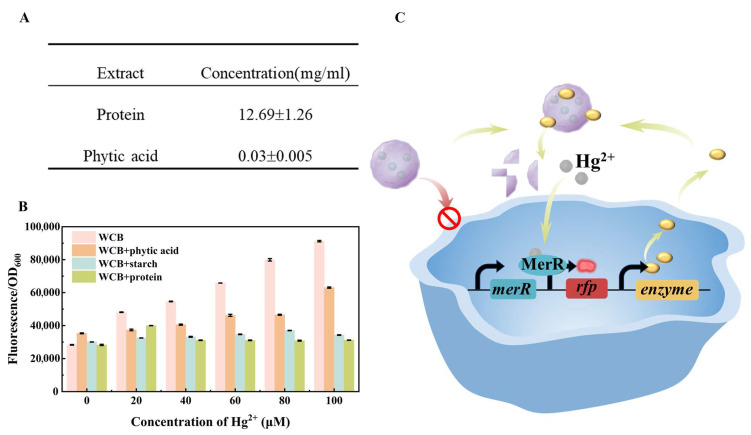
Influence of food matrix on the whole-cell biosensor detection. (**A**) Concentration of the extract. (**B**) Influence of food matrix on the whole-cell biosensor detection. (**C**) Schematic diagram of the “Biological Digestion and Metabolic Pathways”.

Extraction and interference tests were performed on various representative food components to determine which components of organic food matrices most significantly interfere with the accuracy of whole-cell biosensors (WCBs). It is well-established that organic compounds such as phytic acid, starch, and proteins can chelate heavy metals, thereby limiting their bioavailability for detection by WCBs. Based on this concept, we extracted phytic acid from black beans, rice starch, and fish protein (Figure 2A). Phytic acid, starch, and protein were individually introduced into solutions containing varying concentrations of Hg^2+^, and the relative fluorescence intensity of the WCBs was recorded.

Without any matrix component, the relative fluorescence intensity of the WCBs exhibited a strong favorable correlation with Hg^2+^ concentration (R^2^ = 0.9686). The relative fluorescence intensity increased significantly across different concentration ranges, with a detection slope 12,025, indicating that WCBs are highly effective in detecting Hg^2+^ under ideal conditions. However, upon adding organic matrices, the relative fluorescence intensity decreased dramatically, suggesting that organic matrices interfere and adversely affect WCB accuracy. Among the tested components, phytic acid to WCB had a lower influence on relative fluorescence intensity than starch and protein. The relative fluorescence intensity rose with rising Hg^2+^ concentration while maintaining a strong linear relationship (R^2^ = 0.9239), although the detection slope reduced to 4908.3. Starch exerted a more substantial inhibitory effect, lowering the detection slope further to 1031.5. The relative fluorescence intensity increases slowly as the Hg^2+^ concentration increases, causing substantial inaccuracies in results and reducing detection accuracy. Although linear association was maintained (R^2^ = 0.9649), the variations in Hg^2+^ concentration were insufficient to produce distinguishable changes in relative fluorescence intensity. Among all cases, protein exerted the most significant impact on the relative fluorescence intensity. As the concentration of Hg^2+^ increased, the relative fluorescence intensity showed a slight initial increase before leveling off, particularly at concentrations above 40 μM. Under these conditions, WCB’s detection capability was lost entirely, rendering it unsuitable for applications involving protein-rich food matrices (Figure 2B). In conclusion, the presence of phytic acid, starch, and protein significantly interferes with the detection accuracy of the WCB. Therefore, developing an effective strategy to eliminate or mitigate matrix interference is essential for reliable biosensor-based mercury detection.

Phytic acid, which contains six phosphate groups, readily binds Hg^2+^ through electrostatic interactions, followed by a ligand substitution process between Hg^2+^ and H^+^ to form a stable chelate complex [19]. Starch, a natural polymer, exhibits notable adsorption properties. Straight chain starch can physically adsorb Hg^2+^ by producing a single helix shape and applying van der Waals forces. Under acidic conditions, hydroxyl groups on branched-chain starch partially deprotonate and form O-Hg coordination bonds, further enhancing Hg^2+^ binding [20]. In proteins, the sulfhydryl group of cysteine serves as an electron donor, with the sulfur atom forming a stable covalent bond with Hg^2+^ [21]. These interactions are hypothesized to contribute to the reduced detectability of Hg^2+^ in food matrices.

Given the inhibitory effects of phytic acid, starch, and proteins on WCB performance, the bio-digestion and metabolic pathways proposed in Figure 2C were intended to allow matrix-bound Hg^2+^. This approach aims to facilitate cellular uptake of Hg^2+^, thereby minimizing matrix interference and improving detection accuracy.

### 3.2. Screening of Enzymes for the Digestion of Food Matrix Components and Their Impact on Whole Cell Biosensor Performance

**Figure 3:**
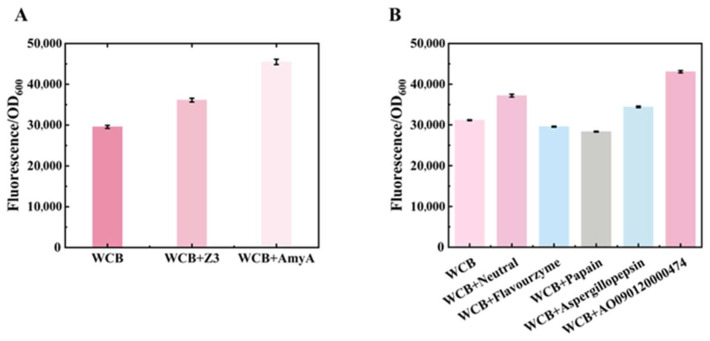
Screening of enzymes for digestion of food matrix components. (**A**) Digestion efficiency of two amylases under Hg^2+^ concentrations of 80 μM. (**B**) Digestion efficiency of five protease under Hg^2+^ concentrations of 100 μM.

To establish an effective bio-digestion strategy, we first evaluated the exogenous addition of organic matrix-digestion enzymes to the detection system to mitigate the interference caused by organic components. Due to its relatively simple structure and stereochemical constraints, phytic acid is only partially digestible by phytase. The commonly used phytase AppA was selected for further experimentation. Two commercially available amylases, Z3 and AmyA, were screened and purchased for starch digestion. These two amylases were mixed and incubated with a detection system that included WCB, starch, and Hg^2+^. Adding Z3 increased the relative fluorescence intensity by approximately 22% compared to the WCB control. However, AmyA demonstrated a more substantial enhancement, improving the signal by 54% of the relative fluorescence intensity. Consequently, AmyA was selected for all subsequent starch digestion experiments (Figure 3A).

To identify a protease with high protein-digestion efficiency, five candidate proteases were evaluated and purchased: neutral protease (Neutral), flavor protease (Flavourzyme), papain (Papain), aspergillopepsin derived from honey fungus (Aspergillopepsin), and a protease derived from *Aspergillus oryzae* (AO090120000474). Each was tested in a co-culture system containing WCBs, proteins, and 100 μM Hg^2+^, Flavourzyme, and Papain, which failed to enhance relative fluorescence intensity and interfered with WCB detection. Aspergillopepsin raised relative fluorescence intensity by 10%, while Neutral increased it by 19%, relative to the WCB control. Notably, AO090120000474 demonstrated a significant improvement, increasing the relative fluorescence intensity by nearly 40% compared to WCBs alone (Figure 3B), showing greater protein-digestion capabilities. Based on these results, AO090120000474 was selected for further use in protein matrix digestion.

### 3.3. Construction of the Biological Digestion Gene Circuit

To reduce the interference of food matrices in WCB Hg^2+^ detection, we selected matrix-degrading enzymes to release Hg^2+^, thereby increasing detection accuracy. While enzyme digestion of food matrices is effective, it complicates the detection procedure and increases operational costs in practical applications. As an alternative, synthetic biology provides a good solution to this problem by loading the genes of the relevant enzymes into a plasmid and constructing a digestion pathway, making the digestion and detection of Hg^2+^ procedure more convenient.

**Figure 4:**
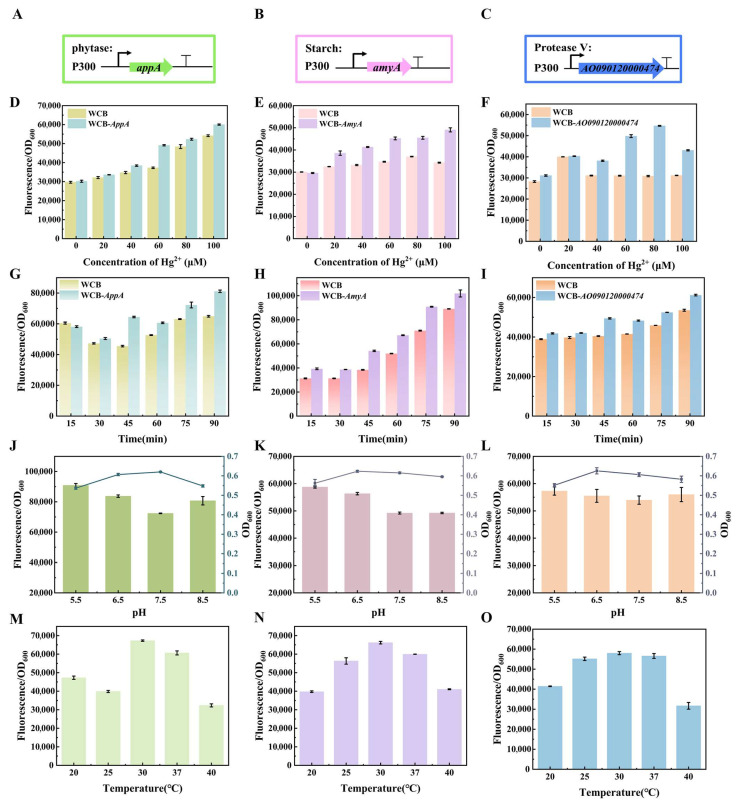
The detection performance and influencing factors of the matrix-digestion whole-cell biosensor. (**A**–**C**) Schematic diagram of the genetic circuit for constructing the whole-cell digestion biosensor. The effect of whole-cell biosensor and WCB-*appA*, WCB-*amyA*, and WCB-*AO090120000474* at different (**D**–**F**) concentrations, (**G**–**I**) time, (**J**–**L**) pH, and (**M**–**O**) temperature.

The *appA*, *amyA*, and *AO090120000474* genes were transferred into the ebMerR-RFP plasmid backbone, resulting in three engineered whole-cell biosensors: WCB-*appA*, WCB-*amyA*, and WCB-*AO090120000474* (Figure 4A–C). Each biosensor demonstrated a concentration-dependent increase in relative fluorescence intensity in response to Hg^2+^. For WCB-*appA,* the detection slope improved to 6166.5 with a 1.11-fold increase in relative fluorescence at 100 μM Hg^2+^ (Figure 4D). WCB-*amyA* exhibited a detection slope of 3486.3 and a 1.43-fold increase in relative fluorescence at 100 μM Hg^2+^ (Figure 4E), indicating restored detection sensitivity in starch-containing systems. Similarly, WCB-*AO090120000474* enhanced detection in protein-rich matrices, with the detection slope restored to 3270.8 and a 1.7-fold increase in relative fluorescence at 80 μM Hg^2+^ (Figure 4F).

The effect of environmental conditions—specifically incubation time, pH, and temperature—is a critical parameter influencing cell survival and metabolism. At 45 min of incubation, WCB-*appA*, WCB-*amyA*, and WCB-*AO090120000474* showed significant increases in relative fluorescence intensity, with absolute signal increments of 18,941.2, 15,742.8, and 8944.43, respectively, compared to WCBs under the same conditions (Figure 4G–I). Given the variety of foods, the pH value of the food being tested can alter cell development, influencing the relative fluorescence intensity of the biosensor. Therefore, it is vital to assess the effect of pH on the detection results of the whole-cell biosensors. WCB-*appA* and WCB-*amyA* maintained reliable detection under mildly alkaline conditions (Figure 4J,K). At the same time, WCB-*AO090120000474* exhibits stable growth and relative fluorescence across the tested pH range (Figure 4L), confirming the robustness of the whole-cell biosensor designs. The fluorescence intensity of the whole-cell biosensors increases significantly at non-optimal temperatures, especially at 30 °C and 25 °C (Figure 4M–O). This phenomenon may be attributed to slower *E. coli*’s proliferation at temperatures below the optimal 37 °C, allowing for prolonged intracellular accumulation of reporter proteins and higher relative fluorescence intensity.

### 3.4. Integration of the Biological Digestion Gene Circuit with Methylmercury Whole-Cell Biosensor

**Figure 5:**
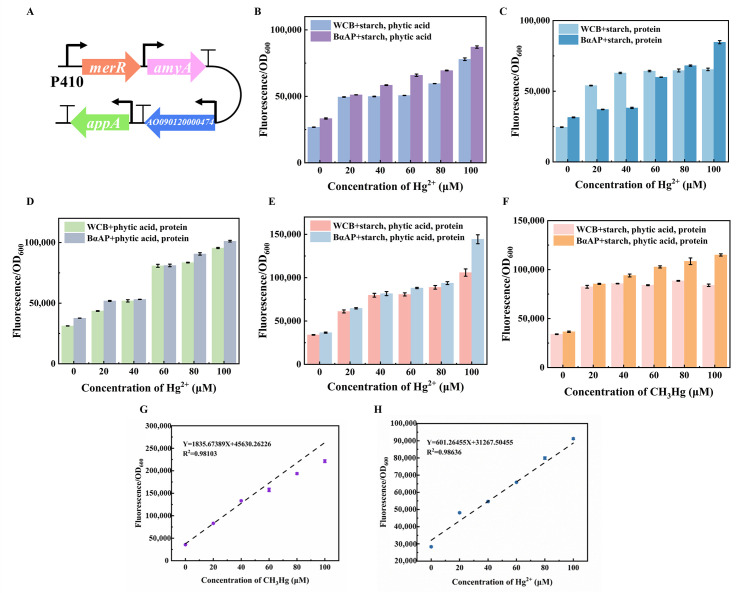
Effect of mixed food matrix on whole-cell biosensor detection. (**A**) Schematic diagram of the genetic circuit for the construction of the BαAP. (**B**) The digestion metabolic pathway is detected when starch and phytic acid are added simultaneously. (**C**) Detection of the digested metabolic pathway when starch and protein are added simultaneously. (**D**) The digestion metabolic pathway is detected when phytic acid and protein are added simultaneously. (**E**,**F**) Detection of the digestion metabolic pathway when phytic acid, starch, and protein are added simultaneously. (**G**) The detection range and linear relationship of CH_3_Hg (LOD = 0.082 μM, LOQ = 0.272 μM, RSD < 1.35%, Detection range = 0–100 μM. Working range = 1–5 μM). (**H**) The detection range and linear relationship of Hg^2+^ (LOD = 0.952 μM, LOQ = 3.174 μM, RSD < 2.91%, Detection range = 0–100 μM. Working range = 1–5 μM).

The cleavage activity of MerB on methylmercury has been demonstrated [16]. To enhance the detection of heavy metals in complex food matrices, the previously tested genes for amylase, phytase, and protease were integrated with the *merB* gene into a single construct to form a synthetic “metabolic pathway for digesting heavy metal pollutants” (Figure 5A). These genes were co-expressed in *E. coli*, creating a digestive module strain called BαAP (comprising *merB*-α-*amylase-appA-protease AO090120000474*). To evaluate the accuracy of BαAP under realistic conditions, a combination of two or three food matrices—starch, phytic acid, and protein—was introduced to simulate real-world sample situations. The biosensor’s accuracy and effectiveness were assessed by measuring changes in relative fluorescence intensity under these conditions. This approach evaluates the functioning and effectiveness of BαAP in complex food matrices, demonstrating its potential application in heavy metal detection scenarios.

When starch and phytic acid were simultaneously present, the relative fluorescence intensity of BαAP increased 1.12-fold at 100 μM Hg^2+^. As the concentration of Hg^2+^ increased, the relative fluorescence intensity of both WCB and BαAP also increased, with the linearity R^2^ for detection effect rising from 0.7498 to 0.9523 (Figure 5B). In samples containing starch and protein, the relative fluorescence intensity increased by 1.29-fold, and R^2^ rose from 0.6388 to 0.9352 (Figure 5C). The presence of phytic acid and protein simultaneously increased the relative fluorescence intensity of BαAP by 1.00-fold compared to WCB (Figure 5D). When all three matrix components—starch, phytic acid and protein—were added concurrently, BαAP displayed superior detection performance. In the presence of 100 μM Hg^2+^, the relative fluorescence intensity increased by 1.36-fold (Figure 5E), while under 100 μM methylmercury, the response improved by 1.37-fold (Figure 5F), demonstrating the biosensor’s ability to handle both ionic and organic mercury forms in complex matrices. In addition, BαAP showed a linear dynamic concentration range from 0 to 100 μM, and limit of detection of CH_3_Hg was 0.082 μM, HgCl_2_ was 0.952 μM (Figure 5G,H).

Simulating food environments covering different types of food showed that the BαAP digestion module can effectively digest various food matrices, releasing Hg^2+^ and mitigating matrix-related interference. Compared to the conventional WCB, BαAP consistently demonstrated higher relative fluorescence intensities and improved detection accuracy. This integrated biosensing-digestion platform establishes a foundation for practical, on-site detection of heavy metal contaminants in real food samples.

### 3.5. Application of the Mercury Whole-Cell Biosensor with Digestion Function in Real Food Samples

**Figure 6:**
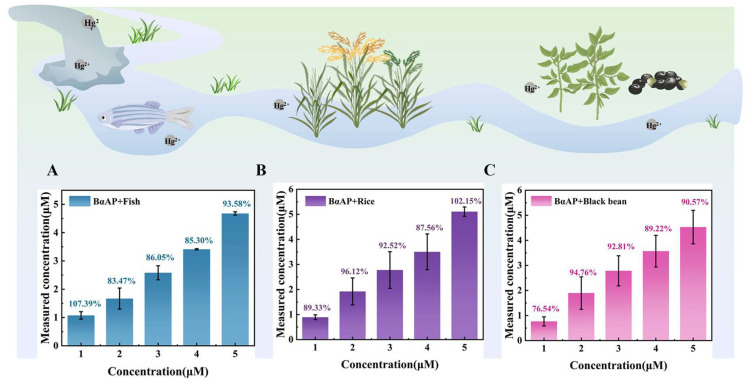
Detection of digestion metabolic pathways in different actual samples. Detection performance of whole-cell biosensors in (**A**) fish, (**B**) rice, and (**C**) black beans after the addition of digestion pathways.

To assess the practical applicability of the digestion pathway in real samples, the BαAP biosensor was used to identify heavy metal concentrations in artificially contaminated food samples. Inorganic mercury (Hg^2+^) can be converted into methylmercury (MeHg) under anaerobic conditions due to industrial emissions and biomagnification. Methylmercury subsequently enters the food chain, accumulating in organisms such as fish and exhibiting high toxicity [22]. To assess BαAP’s detection performance in fish matrices, fish meat samples were artificially contaminated with Hg^2+^ and methylmercury at concentrations ranging from 1 to 5 μM (Figure 6A). Similar tests were conducted using rice—commonly affected by mercury contamination [23] (Figure 6B)—and black beans, a high-protein crop known for its strong matrix effects [24] (Figure 6C). The results showed that Hg^2+^ and MeHg were recovered at rates exceeding 80% across the different food samples (standard curves for mercury and methylmercury are provided in Figure 5G,H). These findings confirm the validity and efficacy of the food matrix digestion and metabolism. Moreover, they demonstrate the effectiveness of the combined digestion-detection whole-cell biosensor in addressing matrix-induced interference, thereby improving the accuracy of heavy metal detection in complex food environments.

## 4. Conclusions

This study developed a bio-digestion technique integrating highly efficient biological enzymes or other biological components into WCB via synthetic gene circuits. This approach replaces traditional physical or chemical sample digestion methods, streamlining detection and improving convenience and accuracy.

Phytase (*appA*) and α-amylase (*amyA*) derived from *E. coli*, along with protease *AO090120000474* from *Aspergillus oryzae,* were linked into WCB to construct whole-cell biosensors for the digestion of phytic acid, starch, and protein, respectively, alongside Hg^2+^ detection. At a Hg^2+^ concentration of 100 μM, the whole-cell biosensors exhibited increases in relative fluorescence intensity of 1.11-fold (phytase), 1.43-fold (amylase), and 1.38-fold (protease), compared to the non-digestion control. Furthermore, environmental tolerance studies demonstrated that all digestion-detection whole-cell biosensors maintained enhanced performance across varied temperature, time, and pH conditions compared with the WCB without the digestion module. Subsequently, the three enzymes were genetically linked to construct a comprehensive “metal contaminant bio-digestion metabolic pathway” to address the complexity of food matrices. The integrated system effectively degraded interfering components when applied to mixed matrices and maintained strong detection performance. The results showed that the established metabolic pathway could effectively digest various components while providing good detection results. Finally, in real food sample applications, the biosensor achieved mercury and methylmercury recovery rates exceeding 80%, confirming the effectiveness and feasibility of the food matrix digestion metabolism and offering a practical solution to the challenge of biosensors’ inaccuracies in heavy metal detection.

## Data Availability

The original contributions presented in the study are included in the article/Supplementary Materials. Further inquiries can be directed to the corresponding authors.

## Supplementary Materials

The following supplementary information can be downloaded at https://www.mdpi.com/article/10.3390/foods14213798/s1, Table S1: Gene sequence used in this study; Table S2: Primer sequence used in this study. Table S3: Plasmid used in this study. Table S4: Gene used in this study. Figure S1: The plasmid map of the WCB + *amyA*. Figure S2: The plasmid map of the WCB + *appA*. Figure S3: The plasmid map of the WCB + *proteaseⅤ*. Figure S4: The plasmid map of the BαAP.
