# Supplementary material for: Replacing Manual Operation with Bio-Automation II: Construction of a Biological Digestion Gene Circuit to Eliminate the Interference of Food Matrices in the Rapid Detection of Heavy Metals
Source: Foods. 2025 Nov 6;14(21):3798. doi: 10.3390/foods14213798 (PMC12609072; doi:10.3390/foods14213798)
Supplement: Supplementary file 1 [file foods-14-03798-s001.zip › foods-3955738-supplementary.pdf]

Supporting information

Replacing manual operation with bio-automation II: Construction of a biological digestion gene circuit to eliminate the interference of food matrices in the rapid detection of heavy metals

Shiqi Xia[1], Shijing Chen[1], Hongfei Su[1], Liangshu Hu[1], Xiaozhe Qi[2, *], Mingzhang Guo[1, **]

1. Key Laboratory of Digital-Intelligence and Dynamic Perception for Food Quality of China Light Industry, Beijing Technology and Business University, Beijing, 100048, PR China

2. Standards and Quality Center of National Food and Strategic Reserves Administration (NAFRA), China, 100834;

***Address correspondence to:** Xiaozhe Qi, Standards and Quality Center of National Food and Strategic Reserves Administration (NAFRA), China, 100834, qxzgrain@outlook.com. Mingzhang Guo, No. 11, Fucheng Road, Haidian district, Beijing, 100048, China, guomingzhang@btbu.edu.cn;

Table S1 Gene sequence used in this study.

Sensor Genes	Nucleotide sequence optimized by this study (5' → 3')
AppA	ATGAAAGCGATCCTGATCCCGTTCCTCTCTTTGCTGATTCCGCTGACACCCCAGTC<br>TGCCTTCGCCCAATCCGAACCTGAGCTGAAACTTGAGAGCGTGGTGATCGTGAGC<br>CGTCATGGCGTTCGCGCACCCACCAAGGCAACCCAACTGATGCAAGATGTGACCC<br>CGGACGCATGGCCGACCTGGCCGGTCAAATTAGGCTGGCTTACTCCTCGTGGTGG<br>CGAACTCATCGCATACCTGGGCCATTATCAGCGTCAACGTCTGGTGGCCGATGGC<br>TTACTTGCGAAAAAAGGTTGCCCCCAATCAGGCCAGGTAGCGATCATTGCCGATG<br>TCGATGAACGCACTCGCAAAACAGGCGAAGCGTTCGCTGCCGGCTTGGCACCAG<br>ATTGCGCAATTACTGTCCATACGCAGGCAGACACATCATCACCGGACCCGCTGTT<br>CAACCCTCTGAAAACGGGCGTATGCCAGCTCGATAATGCCAACGTTACCGATGCT<br>ATTCTGTCACGCGCTGGGGGCTCTATCGCGGATTTCACTGGCCACCGTCAGACGG<br>CCTTTCGTGAGCTGGAACGTGTGCTGAACTTTCCGCAGTCGAACCTGTGCCTGAA<br>ACGTGAGAAACAGGATGAATCCTGCTCGCTTACCCAAGCTCTGCCGAGTGAACTG<br>AAAGTCTCGGCAGATAACGTGAGCCTGACGGGCGCAGTTTCATTGGCAAGTATGT<br>TGACCGAAATTTTCCTGCTGCAGCAAGCCCAGGGCATGCCAGAACCTGGCTGGGG<br>CCGCATCACGGATTCACACCAATGGAATACTCTGCTGTCCCTGCATAACGCACAG<br>TTCTATTTACTGCAGCGCACCCCTGAAGTGGCCCGCAGCCGTGCCACCCCCCTGCT<br>GGATCTTATTAAAACCGCCCTGACACCGCATCCACCACAGAAACAAGCGTATGGC<br>GTTACTTTACCCACCTCAGTGCTGTTCATTGCGGGGCACGATACGAACCTGGCCA<br>ATCTTGGCGGTGCCCTGGAGCTGAATTGGACCTTGCCAGGCCAGCCAGATAACAC<br>GCCACCGGGTGGCGAATTGGTTTTTGAACGCTGGCGCCGCCTGTCGGATAATAGC<br>CAATGGATCCAGGTTAGCCTCGTATTTCAGACCCTTCAGCAGATGCGCGACAAAA<br>CTCCGCTCTCGCTGAATACCCCCCCTGGTGAGGTTAAGCTGACCCTGGCCGGCTG<br>TGAAGAACGTAACGCGCAGGGCATGTGCAGCCTTGCGGGCTTCACCCAGATCGTC<br>AATGAAGCGCGTATTCCGGCGTGCTCCTTATAA
Amylase	ATGAAGCTGGCGGCCTGCTTCTTGACGCTGTTACCGGGGTTCGCCGTGGCCGCGA<br>GTTGGACGAGCCCCGGATTTCTTCTGCCGGGTTTCGCAGTAGCGGCGAGCTTTGT<br>CTCTCATGCACAGCTGCCAAAAGGAACCCGTCCGCTGACCCTTAATTTCGATCAG<br>CAGTGTTGGCAACCTGCAGACGCCATCAAACTGAACCAGATGCTGTCCTTGCAGC<br>CATGCTCTAATACACCGCCACAGTGGCGCCTTTTTCGTGACGGCGAGTACACGCT<br>GCAAATCGACACTCGCAGTGGCACGCCAACGCTGATGATTAGCATTCAAAATGCA<br>GCAGAACCAGTGGCCTCTTTGGTTCGTGAATGCCCGAAATGGGATGGCTTGCCTC<br>TGACCGTGGATGTCAGCGCTACGTTTCCCGAAGGGGCGGCCGTCCGTGACTACTA<br>CAGCCAGCAGATTGCGATTGTGAAAAATGGCCAGATTATGCTGCAGCCGGCAGC<br>GACTAGCAATGGTCTGCTGCTCTTAGAACGCGCGGAAACTGATACATCAGCACCC<br>TTTGACTGGCACAATGCGACCGTGTATTTCGTGTTAACCGACCGTTTTGAAAATG<br>GCGATCCATCGAACGACCAGAGTTACGGTCGCCACAAGGATGGGATGGCAGAAA<br>TCGGAACATTTCACGGCGGGGATCTGCGCGGCCTGACGAATAAACTGGATTACCT<br>GCAACAACTGGGGGTCAACGCGCTTTGGATTAGTGCACCGTTCGAGCAGATCCAC<br>GGTTGGGTTGGCGGACTGTGGAAAGGCGATTTTCCTCACTACGCTTATCATGGCT<br>ACTATACGCAGGATTGGACTAACCTGGATGCTAACATGGGGAATGAAGCGGATC<br>TGCGCACCCTGGTAGACAGCGCACATCAGCGTGGAATTCGCATTCTTTTCGATGT

TGTGATGAACCACACTGGATATGCGACTTTAGCAGATATGCAAGAATACCAATTC
GGAGCGCTCTACCTGTCGGGTGATGAGGTGAAAAAATCGCTGGGAGAACGCTGG
AGCGATTGGAAACCGGCAGCCGGTCAGACTTGGCACTCATTTAACGATTACATCA
ACTTCAGCGACAAAACGGGTTGGGATAAATGGTGGGGCAAGAATTGGATTCGCA
CGGATATTGGCGATTACGACAATCCAGGCTTCGATGACCTGACCATGAGCCTGGC
CTTCCTCCCGGACATTAAAACGGAGAGCACCACCGCGAGTGGCTTACCGGTCTTT
TACAAGAACAAAATGGATACTCACGCTAAAGCAATTGATGGCTATACCCCACGC
GATTACCTGACCCATTGGCTGTCGCAGTGGGTCCGTGATTACGGTATTGATGGCTT
TCGCGTGGATACCGCGAAGCACGTGGAATTACCTGCGTGGCAGCAGTTAAAAAC
CGAGGCGTCAGCAGCGCTGCGTGAATGGAAAAAAGCCAACCCGGATAAGGCCCT
GGATGATAAACCGTTCTGGATGACGGGAGAAGCGTGGGGCCATGGTGTGATGCA
ATCCGATTATTATCGCCATGGATTCGATGCAATGATCAACTTCGATTATCAGGAA
CAAGCGGCCAAAGCCGTGGATTGCCTTGCGCAGATGGACACAACCTGGCAGCAG
ATGGCAGAAAAATTGCAGGGATTCAATGTTCTGAGTTATTTGTCATCTCACGATA
CGCGCTTGTTCCGTGAGGGTGGCGATAAAGCGGCAGAGCTGTTACTGCTTGCCCC
CGGTGCGGTGCAAATTTTCTACGGCGACGAGTCAAGCCGTCCGTTTGGCCCAACC
GGCTCTGATCCTTTGCAGGGCACCCGTAGCGACATGAACTGGCAGGACGTTTCGG
GCAAATCAGCCGCCTCAGTTGCCCATTGGCAGAAAATTAGCCAATTTGGCAAAAG
CGCTGCATCTGTCGCCCATTGGAAGCAAACCACGCTGCTGCTTAAACAAGGCTAC
GGCTTTGTTCGCGAACACGGCGATGATAAAGTGCTGGTGGTGTGGGCCGGACAAC
AATAA

Protease V ATGGTGATTCTGTCTAAGGTTGCCGCAGTTGCAGTTGGTCTGTCCACTGTTGCGTC
TGCGCTGCCAACTGGTCCGAGCCATTCCCCTCATGCGCGCCGTGGTTTCACCATCA
ACCAGATCACGCGTCAGACCGCGCGTGTTGGTCCGAAAACTGCTTCTTTCCCGGC
GATCTATTCTCGTGCACTGGCGAAATACGGTGGTACTGTGCCGGCACATCTGAAA
TCTGCGGTCGCGTCTGGCCACGGCACTGTGGTTACCTCCCCGGAACCGAACGATA
TCGAATATCTGACGCCGGTGAACATCGGTGGCACTACCCTGAATCTGGACTTCGA
CACCGGTTCCGCCGACCTGTGGGTCTTCTCTGAAGAGCTGCCAAAATCTGAACAG
ACTGGTCACGACGTATATAAACCGAGCGGTAACGCCTCCAAGATCGCGGGTGCAT
CCTGGGACATCTCTTACGGTGACGGTAGCAGCGCGTCCGGTGACGTGTACCAGGA
CACCGTAACTGTAGGTGGCGTGACTGCTCAGGGTCAGGCTGTAGAAGCGGCTAGC
AAAATCTCCGACCAGTTTGTCCAGGATAAAAACAACGATGGTCTGCTGGGTCTGG
CGTTCTCTAGCATCAACACCGTAAAACCGAAACCGCAGACCACCTTCTTCGATAC
GGTGAAAGACCAGCTGGACGCGCCGCTGTTTGCAGTGACCCTGAAATACCACGCT
CCAGGCTCTTACGACTTCGGCTTCATCGACAAGTCTAAATTCACCGGCGAACTGG
CCTACGCAGACGTCGACGATAGCCAGGGTTTCTGGCAGTTTACCGCAGATGGTTA
CAGCGTCGGCAAAGGTGATGCACAGAAGGCACCGATCACGGGCATTGCGGACAC
CGGCACCACTCTGGTGATGCTGGACGATGAAATCGTTGACGCCTACTACAAACAG
GTGCAGGGTGCGAAAAACGATGCATCTGCCGGTGGCTATGTATTCCCGTGTGAAA
CGGAACTGCCAGAGTTCACCGTCGTGATTGGCAGCTACAACGCTGTCATCCCTGG
CAAACATATCAACTACGCACCGCTGCAAGAGGGCTCTTCTACCTGCGTGGGTGGC
ATTCAGAGCAACTCTGGTCTGGGTCTGTCTATTCTGGGCGATGTGTTCCTGAAGTC
CCAGTACGTCGTGTTTGACTCTCAGGGTCCGCGTCTGGGTTTCGCTGCGCAAGCA

rfp	ATGGCAAGTAGCGAGGATGTTATTAAAGAATTTATGCGCTTTAAAGTGCGTATGG<br>AAGGCAGCGTGAACGGTCACGAGTTTGAAATCGAGGGTGAGGGTGAAGGTCGCC<br>CGTATGAAGGCACCCAGACCGCCAAACTGAAAGTGACCAAAGGTGGTCCGCTGC<br>CGTTTGCCTGGGATATTCTGAGCCCGCAGTTTCAGTATGGCAGCAAAGCCTACGT<br>GAAGCATCCTGCCGATATTCCGGACTACCTGAAACTGAGCTTTCCGGAGGGCTTC<br>AAATGGGAGCGCGTTATGAACTTCGAGGATGGTGGCGTGGTGACAGTTACCCAG<br>GATAGCAGCCTGCAGGATGGCGAGTTTATCTACAAGGTGAAACTGCGCGGCACC<br>AATTTTCCGAGTGATGGTCCGGTGATGCAGAAGAAAACCATGGGTTGGGAAGCC<br>AGCACCGAGCGCATGTATCCGGAAGACGGCGCCCTGAAAGGCGAAATCAAGATG<br>CGCCTGAAACTGAAAGATGGCGGCCATTACGACGCAGAGGTGAAAACCACCTAC<br>ATGGCCAAGAAACCGGTGCAGCTGCCGGGTGCCTATAAAACAGACATTAAACTG<br>GATATTACCAGCCATAACGAAGACTACACCATCGTGGAGCAGTATGAACGTGCC<br>GAAGGCCGTCATAGTACCGGCGCCTAA

Table S2 Primer sequence used in this study.

**Primer**	**Primer sequence (5' → 3')**
M13-fwd	GTAAAACGACGGCCAGT
M13-rwd	GTCATAGCTGTTTCCTG

Table S3 Plasmid used in this study.

**Plasmid**	Descriptions	Source
pENTR/D- TOPO	Cloning vector, KnR	lab stock

Table S4 Gene used in this study.

**Gene**	**Sequence (5' → 3')**
P300	GGGCGCTTAGACTTTATGCTTCCGGCTCGTATGTTACTCCA
P350	GGTTACTCAGACTTTATGCTTCCGGCTCGTATAATTGAACC
P410	TAGGTATTACACTTTATGCTTCCGGCTCGTATGTTGTTCGC
J23119	TTGACAGCTAGCTCAGTCCTAGGTATAATGCTAG
Shine-Dalgarno	AAGGAGG
T1 terminator	CAAATAAAACGAAAGGCTCAGTCGAAAGACTGGGCCTTTCGTTT<br>TATCTGTTGTTTGTCGGTGAACGCTCTC

T2 terminator	AAAAGGCCATCCGTCAGGATGGCCTTCT

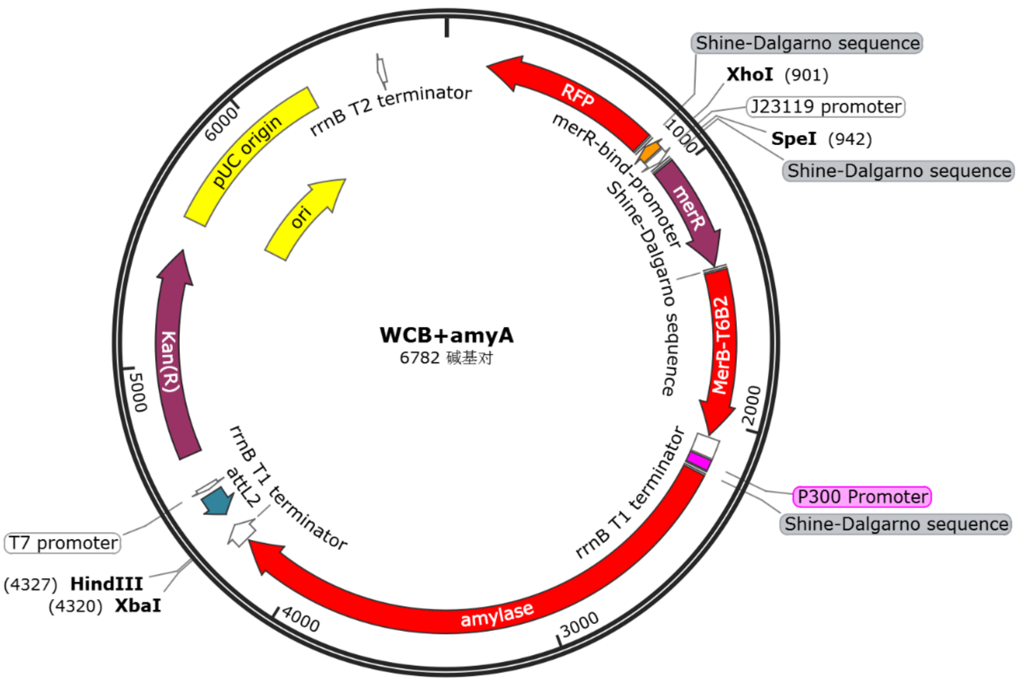

Figure S1 The plasmid map of the WCB + *amyA*.

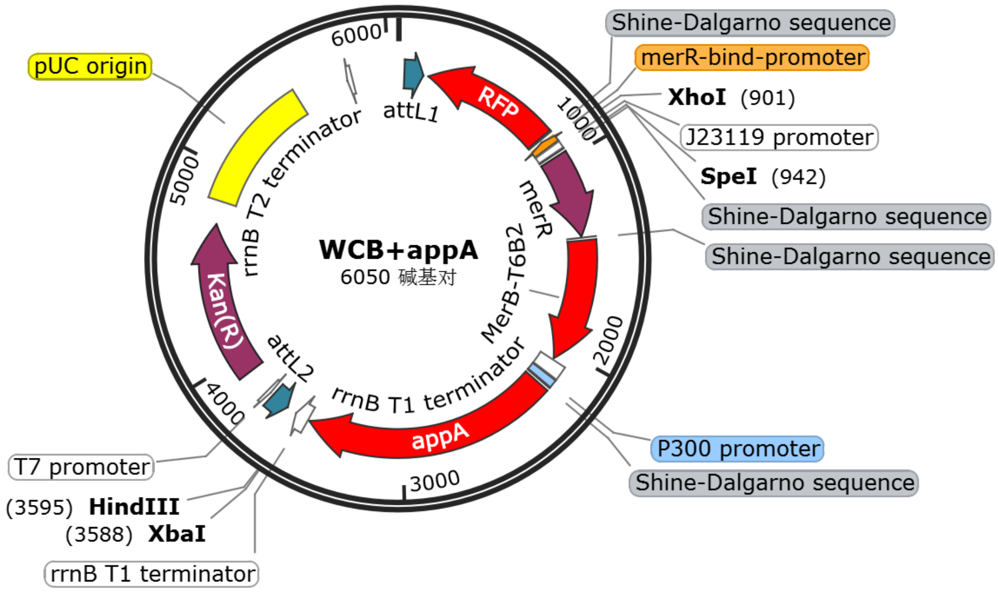

Figure S2 The plasmid map of the WCB + *appA*.

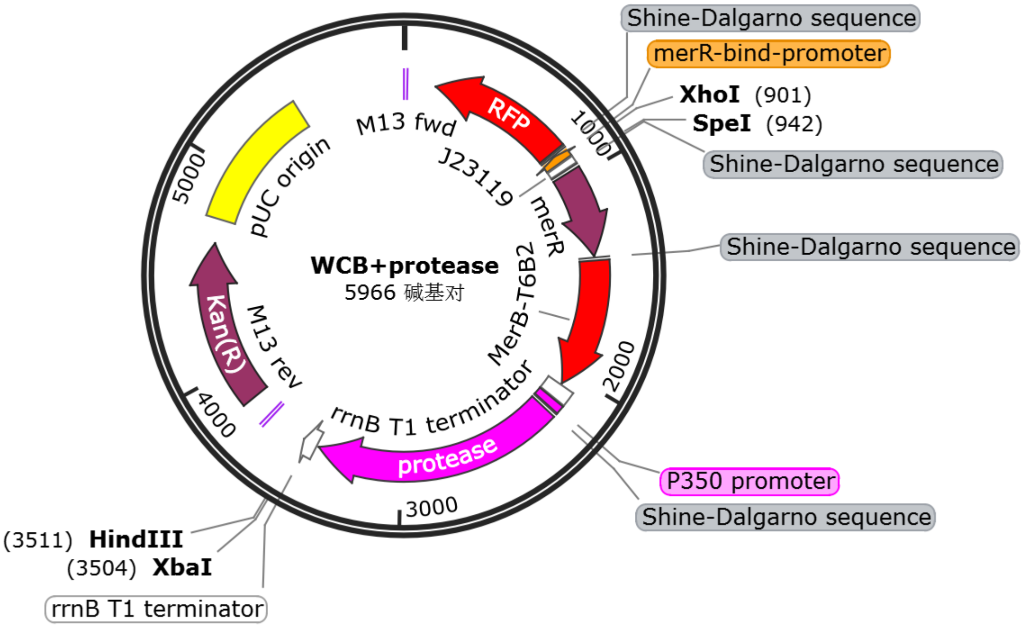

Figure S3 The plasmid map of the WCB + *proteaseV*.

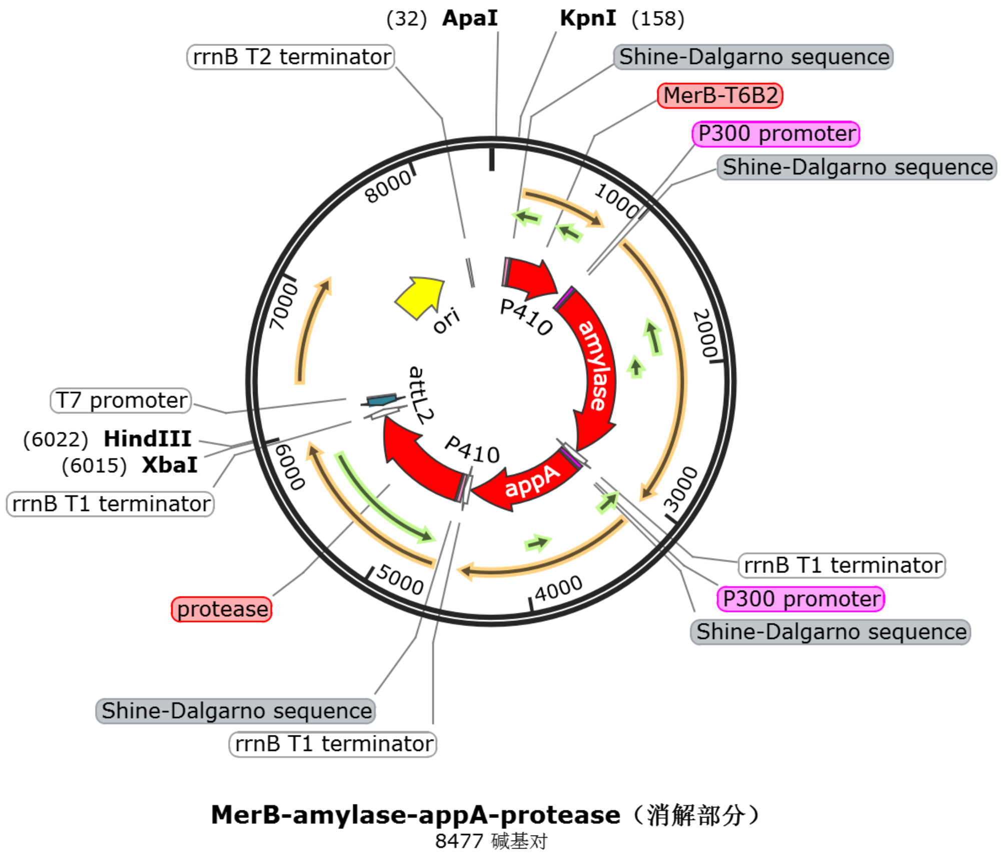

Figure S4 The plasmid map of the BαAP.
